# Membrane-association of mRNA decapping factors is independent of stress in budding yeast

**DOI:** 10.1038/srep25477

**Published:** 2016-05-05

**Authors:** Susanne Huch, Jessie Gommlich, Mridula Muppavarapu, Carla Beckham, Tracy Nissan

**Affiliations:** 1 Department of Molecular Biology, Umeå University, SE-901 87 Umeå, Sweden; 2 Department of Urology, University of Rochester, Rochester, NY 14642, USA

## Abstract

Recent evidence has suggested that the degradation of mRNA occurs on translating ribosomes or alternatively within RNA granules called P bodies, which are aggregates whose core constituents are mRNA decay proteins and RNA. In this study, we examined the mRNA decapping proteins, Dcp1, Dcp2, and Dhh1, using subcellular fractionation. We found that decapping factors co-sediment in the polysome fraction of a sucrose gradient and do not alter their behaviour with stress, inhibition of translation or inhibition of the P body formation. Importantly, their localisation to the polysome fraction is independent of the RNA, suggesting that these factors may be constitutively localised to the polysome. Conversely, polysomal and post-polysomal sedimentation of the decapping proteins was abolished with the addition of a detergent, which shifts the factors to the non-translating RNP fraction and is consistent with membrane association. Using a membrane flotation assay, we observed the mRNA decapping factors in the lower density fractions at the buoyant density of membrane-associated proteins. These observations provide further evidence that mRNA decapping factors interact with subcellular membranes, and we suggest a model in which the mRNA decapping factors interact with membranes to facilitate regulation of mRNA degradation.

Messenger RNA degradation is an important process that modulates gene expression and is essential for normal cellular physiology^1^,^2^. The mRNA degradation occurs in an orderly fashion, beginning with the shortening of the poly(A) tail by the deadenylation complex. After deadenylation, the body of the mRNA can be degraded either from the 5′ or the 3′ end. In *Saccharomyces cerevisiae*, the majority of mRNA degradation proceeds in the 5′ to 3′ direction and is initiated by the mRNA decapping enzyme complex, Dcp1/Dcp2. The decapping enzyme releases the mRNA cap and exposes a 5′ phosphate on the body of the transcript, which allows rapid degradation of the mRNA by the cytoplasmic 5′ to 3′ exonuclease, Xrn1. The mRNA decapping proceeds slowly in yeast without decapping activator proteins, such as Dhh1, Pat1 and the Lsm1–7 complex, which act by either facilitating the activity of the decapping complex or by enhancing mRNA translational repression to promote increased decapping^1^,^3^.

Decapping factors and other proteins involved in mRNA degradation (in addition to mRNA) can accumulate in cytoplasmic aggregates called mRNA processing bodies or P bodies, which have been proposed to be sites of mRNA degradation in the cell^4^. More recently, mRNA decay has been demonstrated to occur concomitantly with translation^5^,^6^. Localised decay either in P bodies or on translating ribosomes could have important implications for how mRNA degradation is regulated. Because decapping dependent decay (i.e., 5′ to 3′ degradation) is the major degradation pathway in yeast after mRNA is deadenylated, we examined the subcellular distribution of mRNA decapping factors to determine the localised decay of mRNA.

Other studies have suggested that mRNA decapping and the 5′ to 3′ decay factors may associate with membranes. Using polysome analyses, both Xrn1 and Lsm7 were found in the most dense fractions^7^,^8^. Several studies have suggested that decapping factors interact with membrane-bound organelles. For example, the mammalian homologue of Dhh1, Rck/p54, was found to be in close association with mitochondria by electron microscopy^9^,^10^. The association with the Golgi apparatus is supported by flotation assays for the mammalian mRNA decay factors, Dcp1a and Xrn1, and by differential centrifugation experiments with Dcp1a^11^,^12^. Similarly, the mRNA decapping activator, Trailer Hitch, which is the *Drosophila* Scd6 homologue, was found using microscopy to be near the exit sites of the endoplasmic reticulum (ER) in granules^13^. Additionally, studies have shown that in both *Drosophila* and yeast, the decapping factor Dcp2 associated with the ER, as determined by a flotation assay and velocity sedimentation^13^,^14^. Finally, the plant Brome mosaic virus (BMV) associates with the ER for its replication and with mRNA decapping factors, which are also critical for BMV replication^15^.

P body resident proteins are ER-associated, including those which form foci due to liquid: liquid phase separation, such as the Whi3 protein^16^,^17^,^18^. Many proteins that have been found in P bodies are associated with the formation of liquid droplets, suggesting that most RNA granules behave like a liquid. However, the behaviour of RNA granules as condensed liquid droplets has not been shown to involve membranes^19^,^20^,^21^. Liquid droplet organelles exhibit a dynamic behaviour that is fundamentally different from membrane-bound organelles. This difference may change the interaction of decapping factors with cytosolic components. The main differences include 1) liquid droplets that are denser than the cytosol and 2) membrane-bound organelles that are less dense. Moreover, the kinetics of association and movement are different^19^. For example, the association kinetics of liquid droplets are dependent on the RNA, and its properties are also determined by the RNA species involved^18^,^22^,^23^. These differences may allow the liquid droplets to more rapidly adjust to changing conditions to alter the RNA metabolism of the cell. Similarly, evidence from yeast and *in vitro* experiments suggest that liquid droplets can develop into a more solid state over time or are intrinsically solid^22^,^24^. When they are less dynamic, solid RNA granules may be less adaptable to rapidly changing conditions.

Here, we found that the decapping factors are distributed throughout the polysomes with special enrichment in the highest density fractions during sucrose density centrifugation. In response to stress, such as glucose deprivation or osmotic stress, this behaviour is maintained. This association is independent of RNA, but it was eliminated with detergent. Furthermore, membrane flotation assays show that the decapping factors migrate to the lowest density fractions similar to proteins that are associated with membranes. Therefore, our data support that the mRNA decapping proteins associate with membranes independent of translation, stress, P body formation or RNA.

## Results

### The mRNA decapping factors are components of larger structures

**Figure 1:**
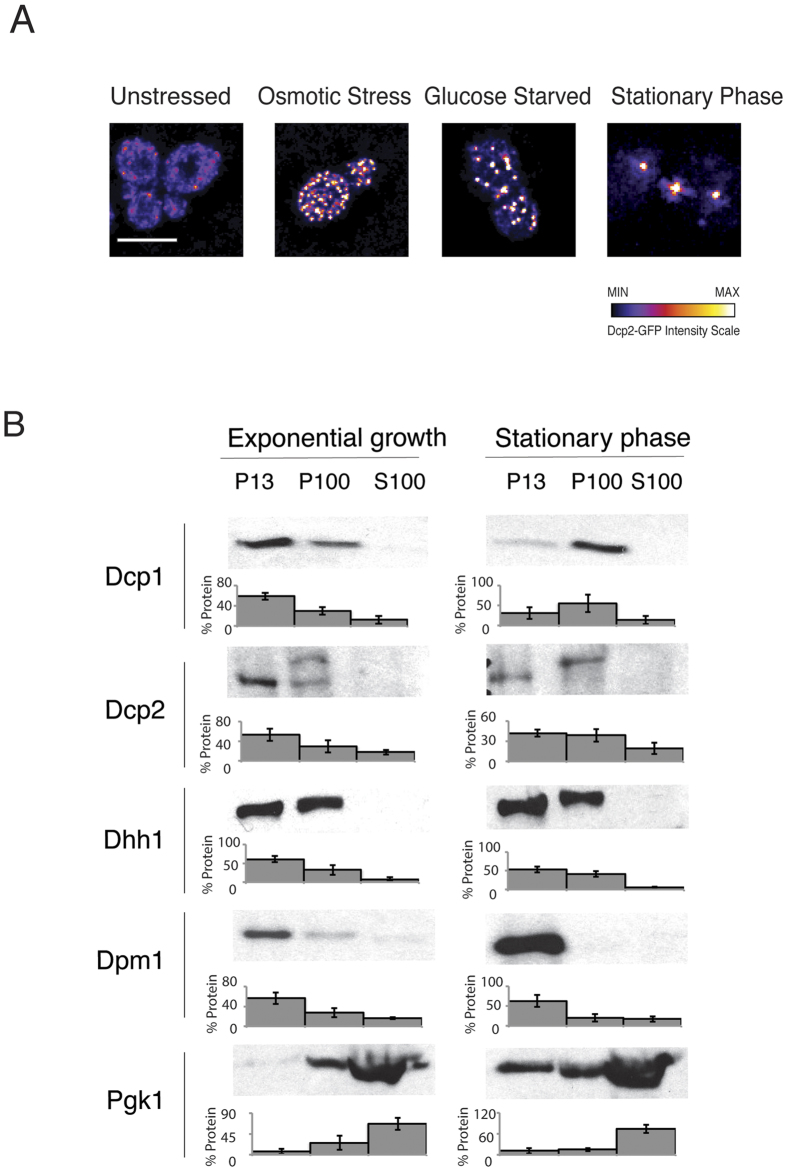
P bodies and differential centrifugation characteristics of decapping factors from unstressed and stressed yeast cells. (**A**) P bodies were observed using DCP2 GFP in exponentially growing conditions. The panels depict the cells in unstressed exponential growth, osmotic stress for 15′ in 1 M KCl, glucose starvation for 15′, or in the stationary phase. All of the panels are adjusted to the same contrast range with the fire lookup table representing the GFP intensity as indicated; the scale bar is 5 μm. (**B**) Western blot of yeast extracts from exponentially growing and stationary phase cells. The strains contain C-terminal TAP tags of the proteins indicated. The extracts were subjected to differential centrifugation to yield a low-speed pellet (P13), a high-speed pellet (P100) and soluble (S100) fractions. Dpm1 is a membrane-associated protein, Pgk1 is cytosolic and Dcp1, Dcp2 and Dhh1 are mRNA decapping factors.

The mRNA decapping factors have been found in P bodies, a type of RNA granule. In unstressed cells, decapping factors are distributed throughout the cytosol and in relatively weak P body foci. Upon cellular stress, P bodies become larger, more numerous and exhibit increased foci intensity^25^. Therefore, we expressed Dcp2 fused to GFP, using the native Dcp2 promoter. We used microscopy to examine the changes in the subcellular distribution of Dcp2-GFP under three stress conditions. These conditions included osmotic stress, glucose deprivation and entry into the stationary phase (Fig. 1A). Compared with the unstressed cells, we found that P bodies were induced by all three conditions: 15 minutes of treatment with 1 M KCl (osmotic stress), after 15 minutes of glucose starvation and when grown to the stationary phase after 72 hours.

Next, we assessed the relative localisation of the decapping factors in the cell using fractionation of *Saccharomyces cerevisiae* extracts to determine if the mRNA decapping factors were associated with P bodies, ribosomes, or other cellular structures. We used differential centrifugation under stressed and unstressed conditions. Differential centrifugation separates cellular components based on size and density, with larger and denser particles pelleting at lower centrifugal forces. To perform these experiments, spheroplasts were generated from mid-log growing yeast cells that contain genomic C-terminal fusion TAP-tagged control proteins and decapping factors that are under the control of their native promoters (Fig. 1B). The extracts were cleared of large cellular debris and centrifuged to generate a P13 pellet, which was enriched in ER, mitochondria, plasma membrane and other large structures. Then, the soluble fraction was subjected to ultra-centrifugation to generate the P100 and S100 fractions, which contain transport vesicles and other smaller membrane structures in addition to ribosomes and soluble protein^26^,^27^.

Based on the previous reports of mRNA degradation occurring in the cytosol, we expected that the mRNA decapping factors would be located predominantly in the P100 or S100 fractions. Surprisingly, the mRNA decapping factors were observed in the P13 and P100 fractions, where membrane structures are enriched (Fig. 1B). The distribution of the decapping enzyme (Dcp1 and Dcp2) and Dhh1 were especially enriched in the P13 fraction and to a lesser extent in the P100 fraction. The P100 fraction can also contain ribosomes, which is consistent with Dhh1 association with polysomes after formaldehyde cross-linking^28^. Thus, these results suggest that mRNA decapping proteins are attached to larger structures and are not free in the cytoplasm. Some such structures could be membranes without ribosomes or membranes that include the ER with associated polysomes^29^.

To examine where *bona fide* membrane and cytosolic proteins localise, we examined extracts for the sedimentation behaviour of the ER protein, Dpm1, and the cytosolic glycolytic enzyme, Pgk1. As expected, Dpm1 was concentrated in the P13 fraction, whereas Pgk1was found predominantly in the S100 fraction (Fig. 1B). Neither Dpm1 nor Pgk1 was significantly present in the P100 fraction. The sedimentation of the decapping factors in the P13 and to a lesser extent in the P100 fraction suggests a membrane association. Next, we examined the same proteins using cells that were harvested during the stationary phase, where P bodies are induced (Fig. 1). We obtained similar results for the control proteins and for Dhh1 as for the mid-log cells. However, the behaviours of the decapping factors Dcp1 and Dcp2 were altered. Dcp1 and Dcp2 exhibited increased sedimentation in the P100 fraction. Intriguingly, we observed an additional band for Dcp2 upon western blotting of the P100 fraction. This additional band could represent a modification of Dcp2 via phosphorylation, as previously reported^30^.

### The mRNA decapping factors sediment in two discrete pools

**Figure 2:**
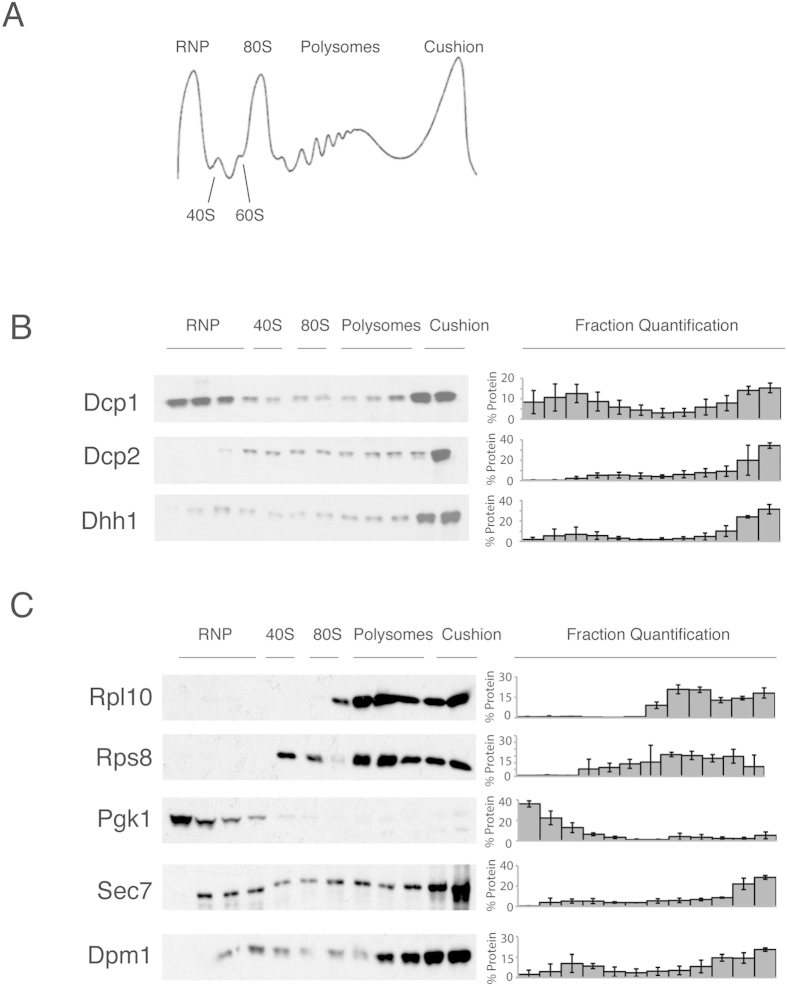
Sucrose gradient distribution of the mRNA decapping factors after ultracentrifugation. (**A**) Typical polysome profile absorbance trace at 254 nm for a 15–50% sucrose gradient, indicating the ribonucleoprotein peak (RNP), ribosomal peaks (40S, 60S, 80S), polysomal peaks and the sucrose cushion peak. (**B**) Western blot analysis of the tagged mRNA decapping protein cell lysates from the 32–55% polysome profile gradient. The localisation of the RNP, 40S, 80S, polysome and cushion regions of the gradient is noted above the western blots. To the right of the blots is the percentage of protein found in each fraction, error = SD, n = 3. (**C**) The Western blot of the untagged cell lysates was probed for Rpl10 and Rps8 protein or lysates from yeast tagged with Pgk1, Sec7 and Dpm1. To the right of the blots is the percentage of the protein found in each fraction, error = SD, n = 3.

Based on our differential centrifugation results, decapping factors are associated with larger subcellular structures or ribosomes. To test these two possibilities, extracts were subjected to velocity sedimentation on sucrose gradients (polyribosome analysis). The extracts were prepared by glass bead lysis and separated on sucrose gradients with an 80% sucrose cushion. This cushion was used to avoid loss of the rapidly sedimenting decapping factors that were observed in the P13 fraction (Fig. 1B). The UV absorbance trace of a standard polysomal 15–50% sucrose gradient exhibited distinct peaks that represent non-membrane-associated ribonucleoprotein complexes (RNP), 40S, 60S and 80S ribosomes and polysomes, and a final peak was observed for the membrane-bound ribosomes at the cushion (Fig. 2A). The RNP fraction consists of RNA and ribonucleoproteins that sediment more slowly than ribosomes^28^,^31^. The large absorbance peak at the cushion is due to the ribosomes in the ER^29^.

To determine if the behaviour of the decapping proteins in the differential centrifugation is due to membrane association, we adopted a modified sucrose gradient protocol that was optimised for the purification of ER membranes^32^. This gradient compresses the ribosomal region of the gradient (Fig. 2B). The decapping proteins concentrate in the densest fractions of the 32–55% sucrose gradient after fractionation, as demonstrated by western blot analysis, with Dcp1 enriched in the low density fractions (Fig. 2B). The decapping activator, Dhh1, and the decapping enzyme subunit, Dcp2, were enriched in the denser fractions within the gradient (Fig. 2B). The localisation of Dhh1 and Dcp2 is consistent with an association with either highly translating mRNA or other large structures. In contrast to Dhh1 and Dcp2, the Dcp1 decapping subunit was concentrated in the most dense and least dense fractions that correspond to the cushion or RNP fractions, respectively (Fig. 2B). The differential localisation of the two subunits of the decapping enzyme complex may be due to the loss of Dcp1 from the complex during centrifugation. Alternatively, it may represent two independent pools of Dcp1. To compare the sedimentation of the decapping factors with that of ribosomes, western blots were probed with antibodies that were specific for the ribosomal proteins, Rpl10 and Rps8, as a reference (Fig. 2C). We further examined the cytosolic enzyme, Pgk1, which localised to the RNP fraction as expected. Next, we used the Golgi protein (Sec7) and ER-associated protein (Dpm1) as markers for the membranes of these organelles^33^. Sec7 and Dpm1 demonstrated similar sedimentation characteristics to Dcp2 and Dhh1 (compare Fig. 2B,C). These data suggest that decapping proteins are associated with either highly translating mRNAs, ER bound ribosomes or other membranes.

### Salt resistance and detergent-sensitivity of rapidly sedimenting decapping factors

Next, we examined whether the rapid sedimentation of the decapping factors is due to their association with membranes or due to their interactions with other large structures, such as the cytoskeleton. To distinguish between these possibilities, we examined the fractionation behaviour of Dcp1, Dcp2 and Dhh1 under three extraction conditions: 1) standard, 2) in the presence of high salt (0.5 M NaCl) and 3) with the addition of Triton X-100. Extracts from these treatments were then separated on a 15–50% sucrose gradient with an 80% sucrose cushion. These conditions distinguish between salt sensitive protein-protein interactions (0.5 M NaCl) and membrane association (Triton X-100).

Cells that were lysed in the presence of high salt were used to determine if the migration of decapping factors in the denser fractions of the gradient were present after stringent lysis^34^. High salt can eliminate ion-dependent protein-protein interactions and remove peripheral membrane-associated proteins. One of the larger structures that the decapping proteins can associate with is the cytoskeleton. A study that systematically purified tagged ORFs, which represent over 80% of the budding yeast genome, found that decapping proteins co-purified with tubulin, supporting the above interaction^35^. Similarly, in yeast, P bodies can contain the tubulin protein, Tub1, and the tubulin ligase protein, Pby1, under certain conditions^36^. Furthermore, high salt has been shown to disassemble the cytoskeleton *in vitro*^37^.

**Figure 3:**
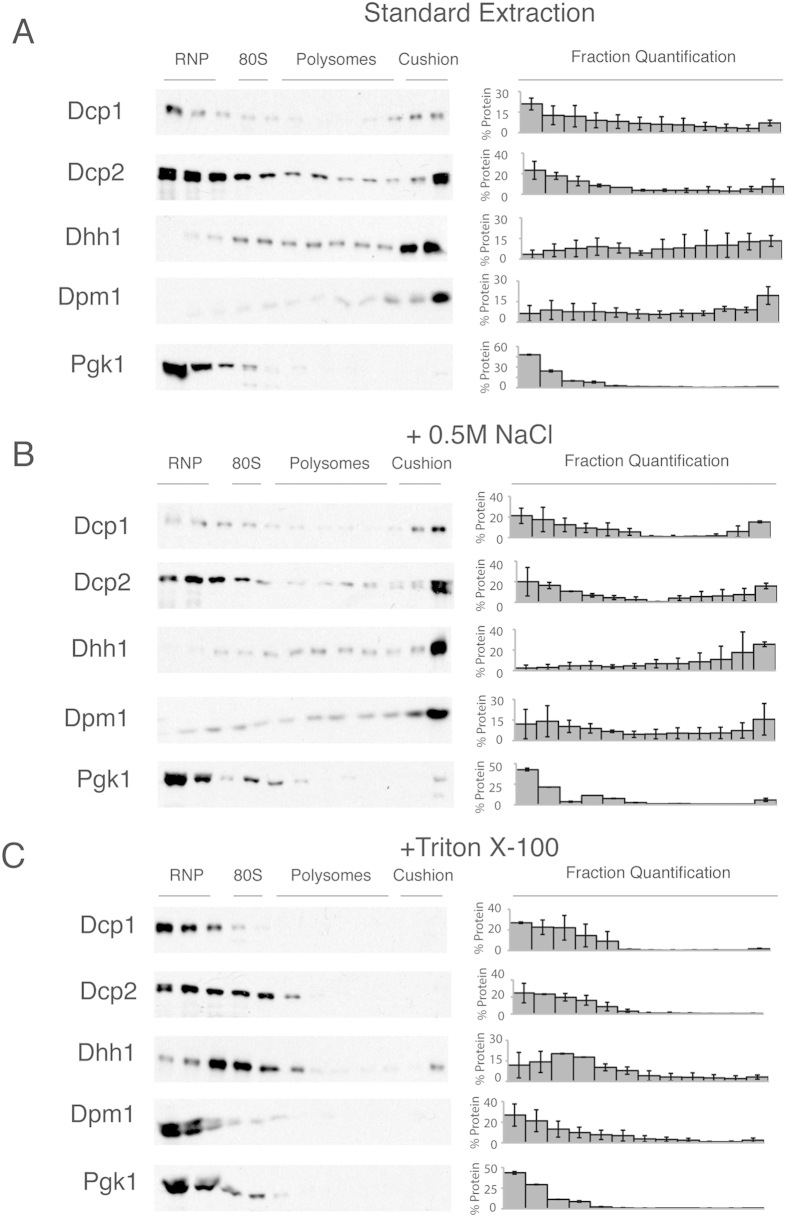
Distribution of the mRNA decapping factors separated on a sucrose gradient under different extraction conditions. (**A**) Western blot analysis of the tagged mRNA decapping proteins from the cell lysate fractions from a 15–50% sucrose gradient. The localisation of the RNP, 40S, 80S, polysome and cushion regions of the gradient is noted above the western blots. To the right of the blots is the percentage of protein found in each fraction; error = SD; n = 3. (**B**) As in panel A, cells lysed with buffer containing 0.5 M NaCl. To the right of the blot is the percentage of protein found in each fraction; error = SD; n = 3. (**C**) Cells lysed with buffer containing 1% Triton X-100. To the right of the blot is the percentage of protein found in each fraction; error = SD; n = 3.

Surprisingly, when we used high salt treatment, all three decapping proteins exhibited similar sedimentation to that observed under standard extraction conditions, suggesting sedimentation is not due to salt-dependent protein-protein interactions (compare Fig. 3A,B). Interestingly, the addition of high salt increases the decapping proteins’ peak in the more dense fractions compared with standard extraction conditions. Because hydrophobic interactions increase in strength in the presence of high salt concentrations, this result is consistent with the membrane-association of decapping factors^38^. We found that high salt has no effect on the membrane or cytosolic controls (Dpm1 and Pgk1, respectively).

Next, we examined whether treatment with detergent could alter the sedimentation of the decapping proteins. To solubilise the membranes, cells were lysed with a buffer that contained the non-ionic detergent, Triton X-100^39^. The decapping factors mostly shifted from the denser fractions of the sucrose gradient near the cushion to the less dense RNP fractions (Fig. 3C). Interestingly, the Dhh1 protein concentration increased in the fraction corresponding to the 40S–80S ribosomal regions under these conditions. We observed this peak in several of our experiments, which is consistent with the association of Dhh1 with the 40S ribosomal subunit^28^. These data suggest that sedimentation in the fractions near the cushion is likely due to association with membranes. The shift from the polysome fraction after lysis with detergent strongly suggests that the rapid sedimentation is not dependent on ER-bound ribosomes because the peak shifted into the RNP fractions, except for Dhh1 as noted above. A more likely possibility may be an association with other membranes. As expected for a membrane-associated protein, Dpm1 also exhibited a shift from the denser fractions to the less dense RNP fractions, indicating sufficient solubilisation of membranes. These experiments support the possibility that the decapping proteins, Dcp1, Dcp2 and Dhh1, are membrane-associated.

### Cellular stress that induces P body formation does not affect the decapping factor migration on a sucrose gradient

**Figure 4:**
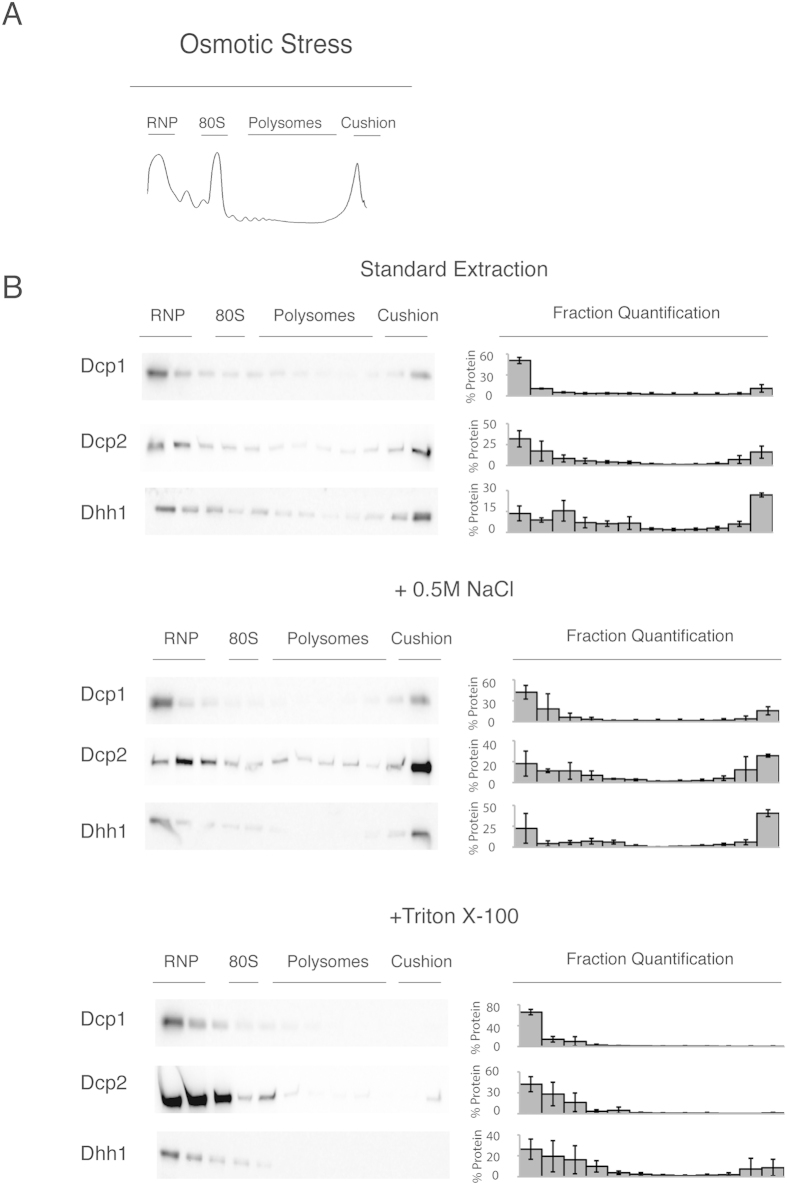
During osmotic stress, the decapping factors were localised to the rapidly sedimenting/dense fractions in the sucrose gradient. (**A**) Typical sucrose gradient profile (at 254 nm) of extracts from mid-log phase of growth, after osmotic stress (15’ treatment with 1M KCl). The extracts were separated on a 15–50% sucrose gradient. The trace demonstrates the RNP, ribosomal peaks (40S, 60S, 80S), polysomal peaks and the peak of the sucrose cushion. (**B**) Western blot analysis of the fractions from the above condition. To the right of the blots is the percentage of protein found in each fraction; error = SD; n = 3. The middle and bottom panels show the tagged mRNA decapping proteins from cells after lysis with 0.5M NaCl or with buffer containing 1% Triton X-100. The localisation of the RNP, 40S, 80S, polysome and cushion regions of the gradient is noted above the western blots.

**Figure 5:**
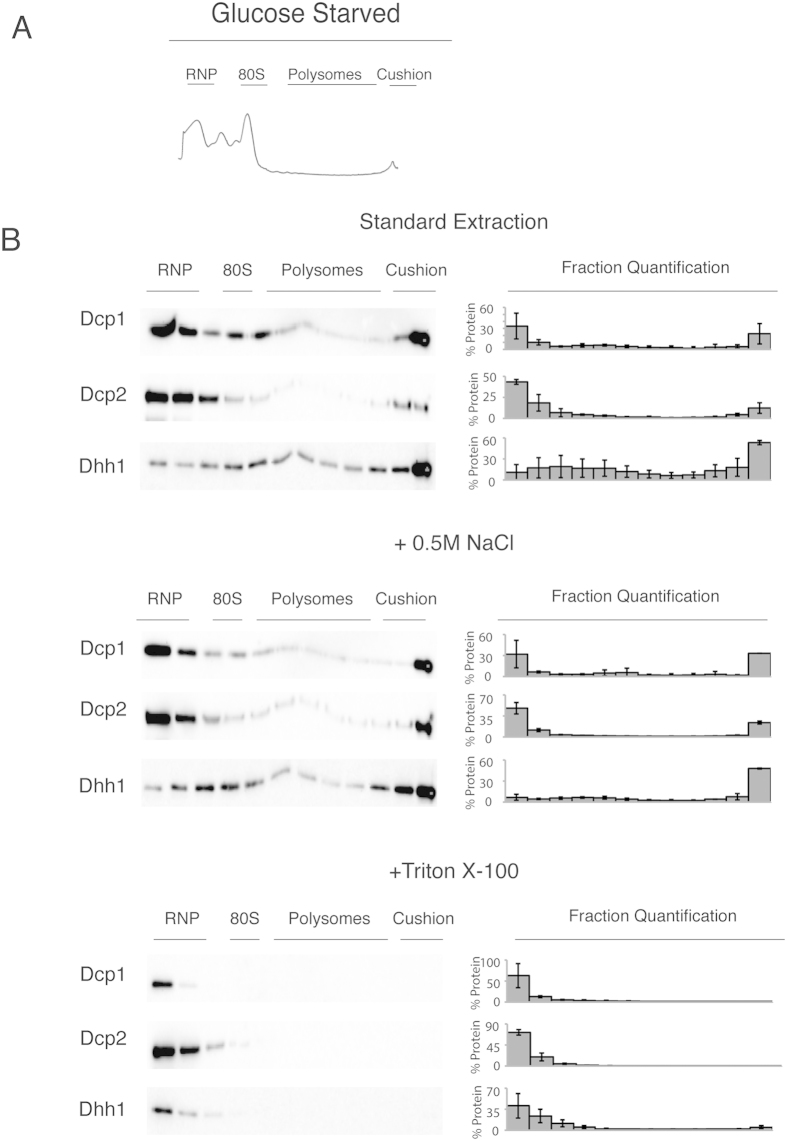
During glucose starvation, the decapping factors localised to dense fractions in the sucrose gradient. (**A**) Typical sucrose gradient profile (254 nm) of extracts from yeast cells growing in mid-log followed by 15 minutes of glucose starvation. The extracts were separated on a 15–50% sucrose gradient indicating the RNP, ribosomal peaks (40S, 60S, 80S), polysomal peaks and the peak at the sucrose cushion. (**B**) The cell lysates from glucose starved cells were then ran on a 15–50% sucrose gradient, and fractions were collected. Western blot analysis of the tagged mRNA decapping proteins isolated from fractions collected from the 15–50% sucrose gradient treated as indicated above the blots. Localisation of the RNP, 40S, 80S, polysome and cushion regions of the gradient is noted above the western blots. To the right of the blots is the percentage of protein found in each fraction; error = SD; n = 3.

The western blots from the sucrose gradients show that the decapping factors behave as if they were attached to a membrane or other detergent-sensitive structure (Fig. 3). An alternative scenario is that the sedimentation profile is affected by the formation of large RNA aggregates, such as P bodies that contain Dcp1, Dcp2 and Dhh1^4^. Therefore, we examined the distribution of the decapping factors during osmotic stress and glucose starvation conditions, both of which induce P body formation (Fig. 1A)^25^. Concomitant with P body formation, osmotic stress and glucose starvation both induced translational repression (Figs 4A and 5A). Because translation is inhibited during osmotic stress and glucose starvation^40^,^41^,^42^,^43^, these results suggest that the localisation of decapping factors in the denser fractions of the sucrose gradient is also independent of translation. Conversely, we examined if cycloheximide would alter the migration of Dhh1 and found that its migration was unchanged (data not shown).

Dcp1-, Dcp2- and Dhh1-tagged yeast strains from the stressed conditions were subjected to standard, high salt and Triton X-100 extractions before separation on a sucrose gradient. The localisation of Dcp1, Dcp2 and Dhh1 in the sucrose gradient was independent of the P body formation that was induced by stress (Figs 4B and 5B). Similar to the unstressed conditions, extracts from stressed cells have decapping factors predominantly in the denser fractions near the cushion of sucrose gradients (Figs 4B and 5B). Additionally, under stress, the sedimentation of the decapping factors is similar to the unstressed conditions. Both were enriched in the RNP and the denser cushion fractions compared with extracts prepared from unstressed cells in both high-salt and standard lysis (compare Figs 3A,4B and 5B). Importantly, with detergent, the decapping factors shift almost entirely to the RNP fraction in extracts from stressed cells, which is similar in behaviour to the unstressed conditions. Taken together, these results suggest that the significantly visible microscopic P bodies do not affect decapping factor localisation in the sucrose gradient.

### Sedimentation of the decapping factors in the dense polysome region of the sucrose gradient is RNA-independent

**Figure 6:**
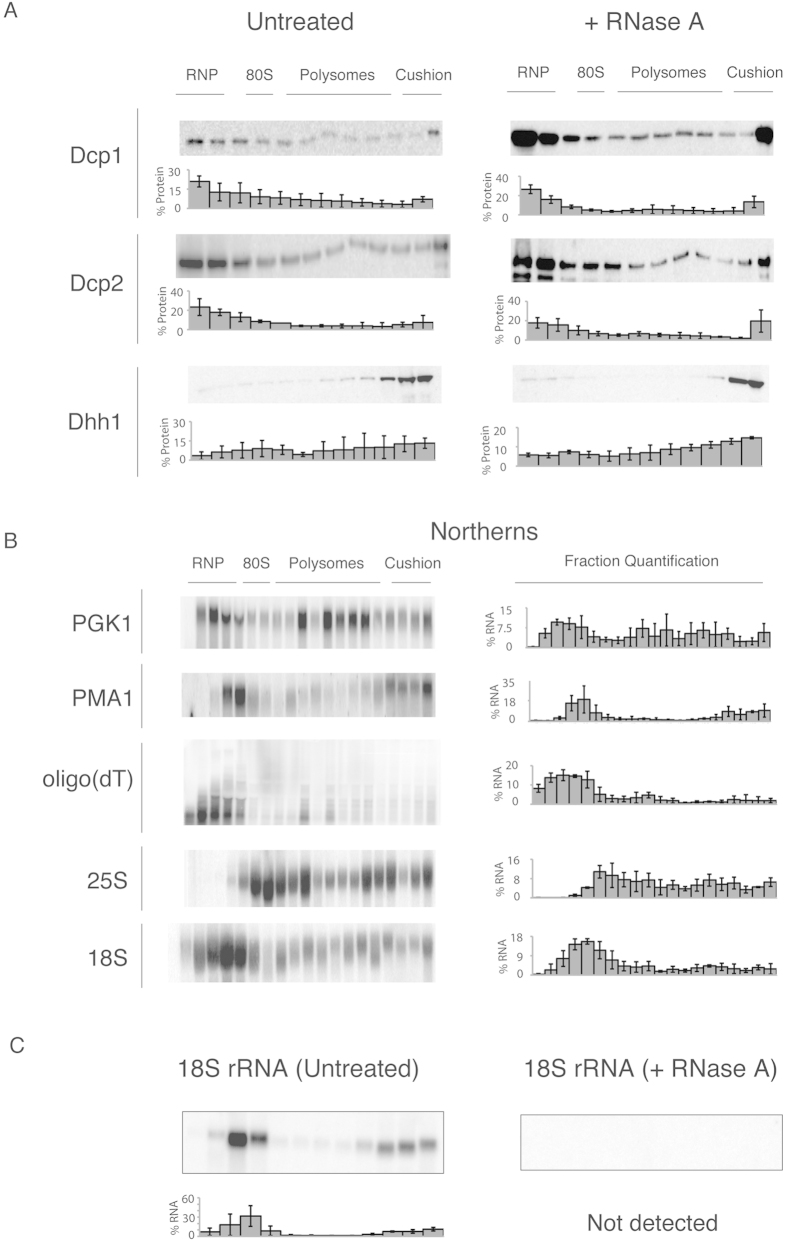
Decapping factor sedimentation in sucrose gradients is RNA independent. (**A**) Western blot analysis of fractions of cell lysates expressing tagged mRNA decapping proteins that were incubated at room temperature for 30 minutes with or without RNase A (1 g/L), followed by separation on a 15–50% sucrose gradient. The localisation of the RNP, 40S, 80S, polysome and cushion regions of the gradient is noted above the western blots. Below each blot is the percentage of protein found in each fraction; error = SD; n = 3. (**B**) Northern blot analyses of the fractions from the sucrose gradients from the lysate of the yeast strain incubated as above and probed for PGK1 and PMA1 mRNA, oligo(dT), 25S rRNA and 18S rRNA. To the right of the blots is the percentage of RNA found in each fraction; error = SD; n = 3. (**C**) As above probed for 18S rRNA in the presence and absence of RNase A. Because no signal was detected in the RNase A-treated sample, it was not quantified.

Our experimental results suggest that decapping factors are associated with large structures that sediment within the dense portion of the gradient (Fig. 2). This association is independent of treatment with high salt and is eliminated by lysis with detergent, suggesting that the sedimentation is due to association with membranes (Fig. 3). Furthermore, the presence of decapping factors in the more dense fractions of the sucrose gradient under stress (when translation is limited) suggests that the localisation of decapping proteins is independent of ribosomes (Fig. 4). Therefore, to further exclude the possibility of the association of decapping factors in the gradient with RNA or ribosomes, we incubated the cellular extract with RNase A prior to ultracentrifugation on a sucrose gradient. The decapping factors persisted in the more dense fractions of the sucrose gradient, similar to the untreated extracts (Fig. 6A). We confirmed the complete digestion of RNA by probing for 18S rRNA (Fig. 6C). These data suggest that the association of the decapping factors with membranes or detergent-sensitive structures is RNA- or ribosome-independent.

### A non-membrane-associated mRNA localises to the translating fractions, whereas a membrane-associated mRNA sediments in the denser sucrose fractions

To examine where mRNA localises within the sucrose gradient, we examined extracts that were separated on a sucrose gradient. RNA was isolated from gradient fractions using phenol/chloroform, followed by agarose northern blotting analysis (Fig. 6B). When harvesting the cells for these experiments, we used cycloheximide to avoid ribosomal run-off^40^. We obtained qualitatively similar results without cycloheximide, albeit with reduced mRNAs in the translating fractions (data not shown). The mRNA encoding for the glycolytic enzyme PGK1 was localised to the 80S and polysomal fractions, with 67% of the mRNA found in these fractions, consistent with active translation (Fig. 6B). Some PGK1 mRNA was localised in the later fractions consistent with being highly translated and its behaviour on sucrose gradients^44^. Then, we probed the blot for PMA1 mRNA, which encodes for a trans-membrane protein and should therefore be associated with the rough ER. This mRNA was enriched in the cushion/membrane-associated fractions, with 36% found in the five densest fractions. In comparison, 20% of the PGK1 mRNA was found in these fractions (Fig. 6B). These results were confirmed by probing for oligo(dT) to identify the localisation of poly(A)+mRNA. We found a large proportion of lower molecular weight poly(A) +RNA in the RNP fractions. These may represent polyadenylated products from the rRNA, tRNA, snRNA degradation pathways^45^,^46^,^47^. To confirm translation, we probed for the 25S and 18S rRNA to identify the localisation of the ribosomes in regards to the mRNAs. Finally, to examine digestion by RNase treatment, we treated extracts with RNase or with no RNase and separated them on a sucrose gradient. The 18S rRNA was enriched predominantly in the third fraction, which corresponds to the 80S ribosome (Fig. 6C). Additionally, a portion of the 18S rRNA was identified in the fractions near the sucrose cushion. We performed the same experiments with RNase A-treated extracts and analysed them using northern blotting. We did not observe any 18S rRNA on the blot, despite the same exposure time and contrast range as for the northern blot of the untreated extracts (Fig. 6C).

### The mRNA decapping factors are membrane-associated

To more directly distinguish between proteins in low-density membrane fractions and those in high-density protein aggregates, we performed a subcellular fractionation protocol based on membrane flotation analysis of the lysates. The extracts of tagged mRNA decapping factors were subjected to equilibrium centrifugation to reach their buoyant density in high-density OptiPrep (iodixanol) gradients via ultracentrifugation^48^. Proteins that are not associated with membranes have a high density, whereas membrane-associated proteins should float due to association with less dense lipids^49^.

**Figure 7:**
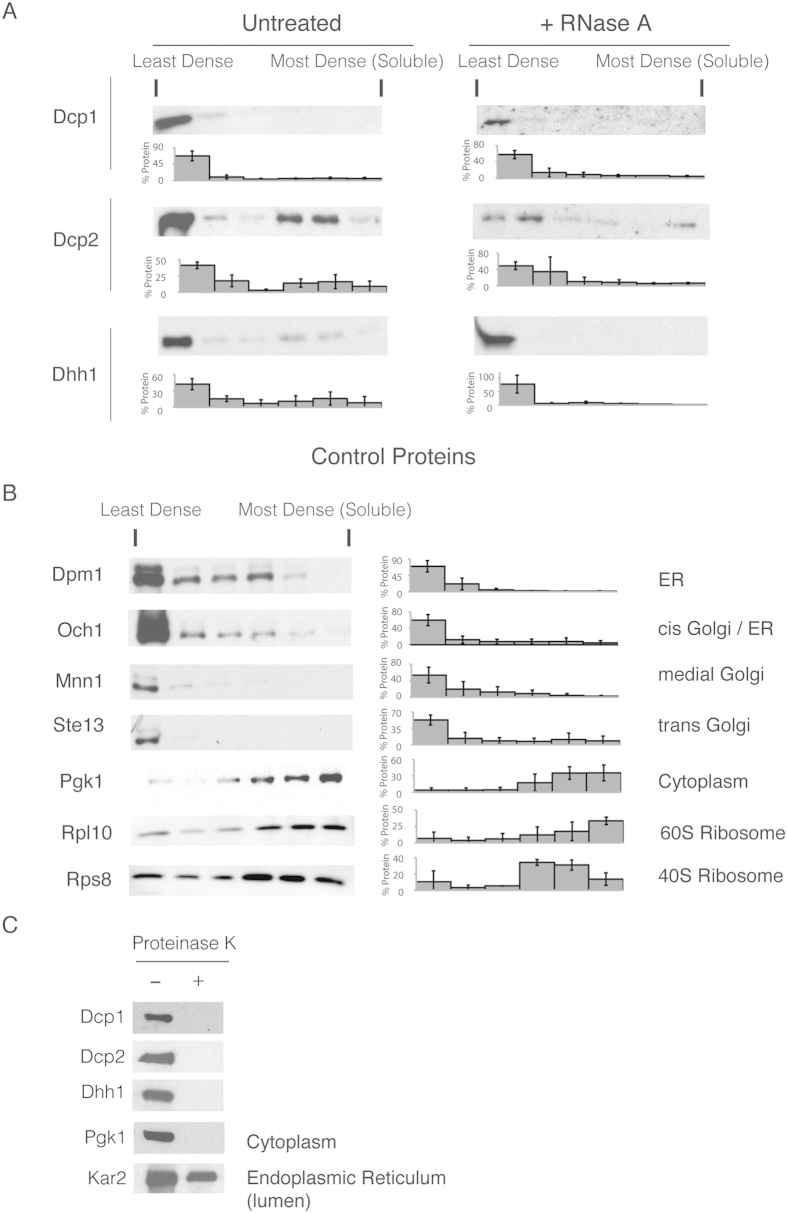
Buoyant density distribution of mRNA decapping factors and control proteins in flotation assays. Western blots of fractions from the OptiPrep flotation assay of tagged (**A**) mRNA decapping proteins or (**B**) control proteins. To the far right of the western blots are the control proteins’ subcellular localisations. The less dense portion of the gradient is indicated by “Least dense”, and the dense bottom portion is labelled as “Most dense (soluble)”. To the immediate right of the western blots is the amount of protein in each fraction; error = SD; n = 3, except for Och1, Mnn1, and Ste13 where n = 2. (**C**) Yeast extracts of the indicated strains that expressed the C-terminal TAP tag fusions were incubated with lysis buffer (−) or digested with Proteinase K (+) for thirty minutes at room temperature.

To perform the membrane flotation assays, yeast spheroplasts were generated and lysed with a Dounce homogeniser because glass-beads can be damaging to this assay^26^. The lysis of spheroplasts, however, precludes the possibility of examining multiple stress conditions during these experiments. We examined the decapping factors, Dcp1, Dcp2 and Dhh1, all of which floated in the least dense portion of the OptiPrep gradient, suggesting that they are associated with membranes (Fig. 7A). The results from the polysome analysis/sucrose gradients suggest that this localisation is not RNA-dependent (Fig. 6A). To further confirm this result with the flotation assay, we examined the flotation of the proteins after RNase A treatment (Fig. 7A). Our results suggested that the decapping factors associate with membranes independent of the RNA (Fig. 7A). Lysates with tagged proteins that associated with the endoplasmic reticulum (Dpm1), Golgi apparatus (Och1, Mnn1 and Ste13) or cytosol (Pgk1) were also examined (Fig. 7B). The specific localisation of the Golgi markers is indicated on Fig. 7B, as previously described^33^. The ER and Golgi proteins floated in the lower density portions (Fig. 7B). In contrast, Pgk1, which is not membrane-associated, was more concentrated in the densest fractions. Finally, consistent with the 18S localisation on polysomes, the ribosomal proteins, Rpl10 and Rps8, were mostly found in the denser fractions with a portion floating on the gradient (Compare Figs 6B and 7B).

These data suggest that all of the mRNA decapping proteins that were examined are membrane-associated. However, we cannot exclude the possibility that the mRNA decapping factors associate with membranes during or after cell lysis. In addition, it is possible that most of these proteins are not themselves directly membrane-associated. The exact proteins that provide a direct contact with the membranes are unknown, and we have not yet been able to identify them by examining the behaviour of extracts of mutant strains that were deleted as potential mediators for membrane interactions with the decapping complex (data not shown). Furthermore, because these proteins are involved in multiple mutually interacting complexes, the primary membrane-associated protein(s) may be difficult to identify.

### The mRNA decapping factors are accessible to the cytosol

If mRNA decay factors are membrane-associated, they may be located within the membranes. We examined this possibility using an assay that relies on membranes to protect the protein from degradation by proteinase K^50^. After lysis of mid-log phase cells and clearing of the debris, half of the lysate was treated with proteinase K, and the other half was left untreated. Following incubation, the lysate was precipitated with TCA and analysed using western blotting. Our control, the cytosolic protein, Pgk1, was fully digested as expected (Fig. 7C). In contrast, Kar2, which is present in the ER lumen, was largely protected. All of the mRNA decapping factors that were examined were sensitive to proteinase K treatment, similar to the behaviour of Pgk1. These results suggest that the mRNA decapping proteins are accessible to the cytosol and are not enclosed in membranes.

### Microscopic localisation of decapping factors to membranes

**Figure 8:**
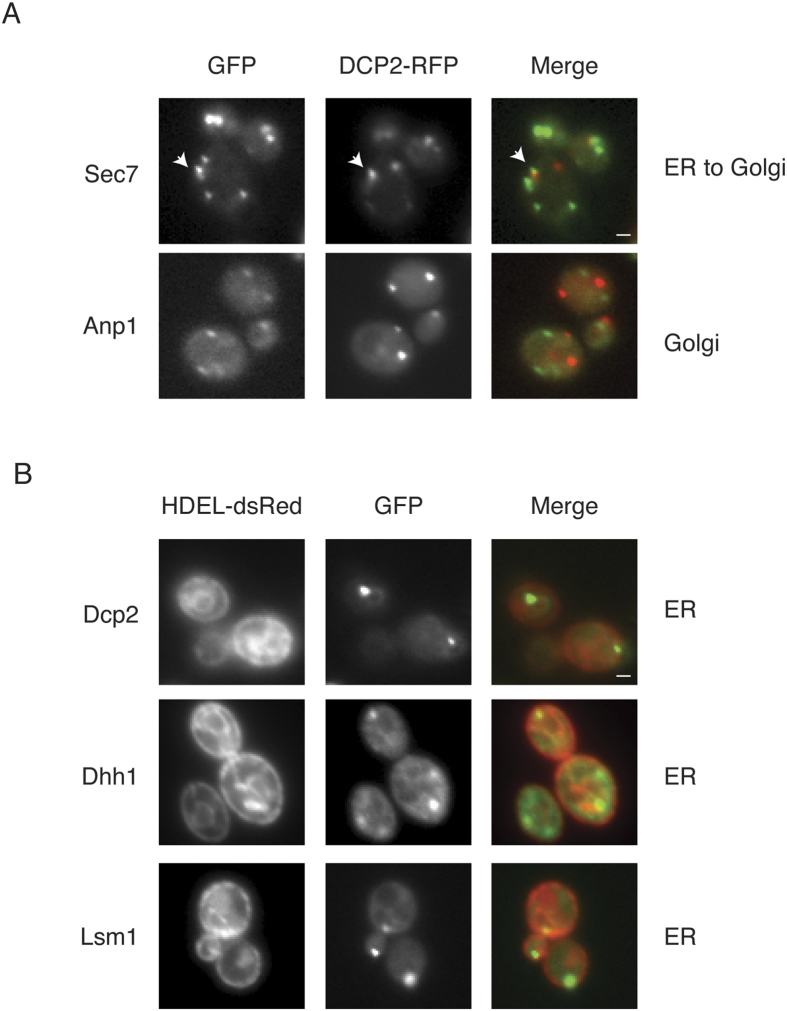
The decapping factors are adjacent or co-localised with the Golgi apparatus and/or ER. (**A**) The exponential-phase cells that expressed chromosomal GFP-tagged proteins (indicated to the left) that expressed Dcp2-RFP on a plasmid were glucose deprived for 15 minutes. The GFP-tagged protein localisation is indicated to the right. An arrow indicates the adjacent Dcp2 and Sec7 foci; the scale bar is 1 μm. (**B**) Exponential-phase cells that expressed both chromosomal GFP-tagged decapping proteins (indicated to the left) and chromosomally integrated HDEL-dsRed were subjected to 15 minutes of glucose deprivation. The localisation of HDEL-dsRed is indicated to the right; the scale bar is 1 μm.

Because decapping factors are membrane-associated, we examined their relationship to the Golgi apparatus using microscopy of fluorescently tagged Golgi proteins (Sec7 and Anp1) and an ER marker, HDEL-dsRed^51^. Cells were stressed by glucose deprivation to induce P body formation to aid in the co-localisation. The Golgi protein, Anp1, did not co-localise with Dcp2-mCherry (Fig. 8A). A modest 15% of the Dcp2 foci were adjacent to or co-localised with the ER to the Golgi protein, Sec7 (Fig. 8A). In contrast, 79% of the Dcp2 foci were adjacent to or co-localised with the ER marker protein, HDEL-dsRed (Fig. 8B), similar to the ER-associated protein, Cln3, which forms cellular foci of RNA and protein^16^,^18^. We examined two more decapping activator proteins, Dhh1 and Lsm1, which are also adjacent to or co-localised with the ER marker, HDEL-dsRed, in 88 and 84% of their foci, respectively (Fig. 8B). These results suggest that the decapping factors are localised at or near the ER or Golgi, which is consistent with previous microscopy experiments^13^,^14^.

## Discussion

We combined several approaches to examine the polysomal and subcellular localisation of mRNA decapping factors, which are the core components of P bodies. Here, we provide evidence that the mRNA decapping factors are membrane-associated, independent of stress or the RNA (Figures 2, 3, 4, 5, 6, 7, 8). Ultimately, the effect of membrane-association of mRNA decay factors on mRNA degradation is uncertain, although we envision three non-mutually exclusive scenarios.

First, membranes can function as a storehouse for mRNA degradation factors, allowing mRNA to be protected until degradation or translation is signalled. Membrane-associated decay factors may facilitate regulation of mRNA degradation, either via promotion of degradation or via inhibition of decay. Because the mRNA decapping factors are concentrated in P bodies under multiple stresses, P bodies may serve as a convenient way station for mRNA entering and leaving translation on the ER, and they may serve as a source to feed selected mRNA from repression into localised translation on the ER.

Second, the mRNA decay factors may be spatially separated to facilitate regulation of mRNA degradation. This spatial separation between membrane-bound factors and the cytosolic RNA may allow key factors to remain soluble to prevent precocious mRNA decapping and subsequent degradation. The nature of this regulation will require identification of the protein(s) that direct the factors to the membranes.

Third, messenger RNA degradation may occur on membranes, which is an emerging concept in prokaryotes^52^. For example, the enzymes that are involved in the major pathway of mRNA decay in *E. coli* (RNase E and the degradosome) are localised to the cytoplasmic face of the plasma membrane^53^, and their localisation can affect mRNA decay^54^. Additionally, one of the major mRNA degradation enzymes in *Bacillus subtilis*, RNase Y, has been linked to membranes^55^,^56^. A similar phenomenon may be found in eukaryotes because multiple reports have tied siRNA and miRNA repression to various membranes^12^,^32^,^57^,^58^. There may be a complex relationship between mRNA decay and membrane-association in eukaryotes, which is still undefined.

## Methods

### Yeast Plasmids, Strains, and Cell Growth

The genotypes of the strains used in this study are listed in Table 1. Yeast strains were grown on either standard yeast extract/peptone medium (YEP) or synthetic medium (SC) supplemented with the appropriate amino acids and 2% glucose or 2% galactose as indicated. The plasmids used in this study were pRP1155 expressing full length Dcp2 fused to RFP^25^ and pKW1803, an integration plasmid expressing the ER/NE luminal marker HDEL fused to dsRed^51^. To test for glucose depletion and osmotic stress, exponentially growing yeast were centrifuged, washed in appropriate media (either lacking glucose or made with addition of 1 M KCl) and re-suspended in the appropriate media. After the indicated growth time with aeration, the cells were harvested.

### Live Cell Microscopy

Live yeast cells were re-suspended in appropriate minimal media and visualized on a Deltavision Spectric microscope with an Olympus 60 × 1.4NA objective without binning unless otherwise indicated. Deltavision microscopic images were deconvolved using the classical maximum likelihood estimation algorithm in Huygens Essential 4.4 (SVI). The resulting image was depicted by maximum intensity projections of a Z series of 20 slices of 0.25 μm thickness displayed with Fiji^59^. Projections were in the same contrast range and displayed by using the fire lookup table. Glucose deprivation stress was conducted as previously described for exponentially growing cells (OD600 of 0.3 to 0.5)^60^. Co-localisation experiments were performed on a Nikon Eclipse 90i with a Nikon 60 × 1.4NA objective without binning. Co-localisation images are presented as single plane data and analysed as such to optimize co-localisation accuracy. At least 50 cells were counted for each panel. Co-localisation of mRNA decapping factors was assessed by visual inspection. The numbers of decapping factor foci in the cells were counted. They were considered as co-localised when the factors superimposed with ER or Golgi marker protein fluorescence. They were considered adjacent when the Golgi/ER fluorescence was less than ~100 nm from the respective decapping factor but not overlapping. The identity of the mRNA decapping factor and the membrane marker protein was blinded during scoring.

### Western Blot Analysis

Western Blots were detected with either Peroxidase-Anti-Peroxidase (Dako or Sigma) at 1:2500 or TAP Tag Antibody (Pierce) at 1:2500. The Rps8 and Rpl10 antibodies were used at 1:5000 dilution and were detected using goat anti-rabbit HRP (Agrisera).

### Subcellular Fractionation

Subcellular fractionation was performed using differential centrifugation as described^26^. Briefly, yeast spheroplasts were prepared from 15 A600 units of cells re-suspended in 1x TN buffer (50 mM Tris-HCl, pH 7.4, 150 mM NaCl, 1 mM DTT). Cells were lysed with a Dounce homogenizer and the lysate was cleared by centrifugation in a microcentrifuge at 1,500 g for 5 minutes at 4 C. Afterwards, the lysate was centrifuged at 13,000 g at 4 C in a microcentrifuge to generate the P13 pellet. The supernatant was removed for the subsequent steps and the pellet was gently overlaid with lysis buffer and centrifuged again, discarding the second supernatant. The pellet was re-suspended in lysis buffer with 0.1% SDS for further processing as P13. The supernatant from the initial centrifugation was then centrifuged in a table-top ultracentrifuge using a TLA110 rotor at 100,000 g for 60 minutes. The supernatant was retained for further analysis (S100) and the pellet was processed as above (P100). The P13, P100 and S100 fractions were TCA precipitated and analysed by western blot.

For proteinase K treatment, lysis was performed as above. The cleared extract was separated in two fractions. Half of the lysate was digested with 0.2 g/L proteinase K (Fisher) and half left untreated, with both incubated for equal time at room temperature followed by TCA precipitation. The untreated portion was incubated at room temperature for an equivalent time without proteinase K and then precipitated with TCA.

### Sucrose Gradient Sedimentation

Sucrose gradient analysis was performed similarly to the method previously described^20^. Cells were grown as indicated above. Harvesting was with centrifugation at room temperature for 1 minute at 3000 g. Cells were lysed at 4 C using glass beads with incubation on ice for 5 minutes after 2 minute pulses in a Disrupter Genie with 1x TN buffer supplemented with and Complete EDTA Free Protease Inhibitor (Roche). Lysis buffer was supplemented with either 0.5 M NaCl or 1% Triton X-100 as indicated. Cycloheximide was not used for protein analysis during cell harvesting due to its ability to eliminate P bodies^4^,^61^. For RNA analysis, the cells were harvested at 4 C on ice supplemented with 2 ml of 10 mg/ml cycloheximide due to the larger volume of cells and lysed with 0.5 mg/ml heparin and 10 mM ribonucleoside-vanadyl complex added to the lysis buffer (see text)^40^. Cellular debris was cleared by centrifugation at 1,500 g. The resulting supernatant was loaded on to either a 32–55% or 15–50% sucrose gradient with an 80% sucrose cushion as indicated. After ultracentrifugation at 39,000 rpm for 90 minutes, A254 was monitored using a continuous flow cell UV detector (Isco).

### RNA Analysis

RNA was purified from individual fractions of the sucrose gradient after incubation at room temperature with or without RNase A (at 1 mg/ml concentration). RNA was extracted with Phenol/Chloroform/Isoamyl Alcohol (Ambion) and ethanol precipitation. RNA was separated on agarose gels and followed by northern blotting for 25S, 18S rRNA, oligo(dT), PMA1 and PGK1 mRNAs and oligo(dT) by and detected with oTN 194(CATGGCTTAATCTTTGA GAC), oTN198 (CATGGCTTAATCTTTGAGAC), oligo(dT) 15 primer (Promega), oTN747 (GCTTCACCGGCGGCAACTGGACCATCG) respectively or random primed PCR fragment of genomic DNA amplified using oTN272 and oTN273 (TTGTCTGTCCAAGATTTGGACT and AAGAAAGCAACACCTGGCAA) for PGK1.

### Membrane Flotation Assay

The assay followed the previously published procedures^48^. Briefly, yeast spheroplasts were prepared from 15 A600 units of cells re-suspended in 1x TN buffer. Cells were lysed with a Dounce homogenizer and the lysate was cleared by centrifugation in a microcentrifuge at 1,500 g for 5 minutes at 4 C to remove cellular debris. 250 μl of the resulting supernatant was mixed with 500 μl 60% OptiPrep iodixanol (Axis-Shield). 600 μl of this mixture was added to tube and filled with 1.4 ml 30% OptiPrep and overlaid with 100 μl lysis buffer. The samples were centrifuged for 2 hours in a Beckman SW-60 or a TLA100.3 rotor (54,000 rpm). After centrifugation, the samples were either boiled in Laemmeli buffer or precipitated with TCA.

## Additional Information

**How to cite this article**: Huch, S. *et al*. Membrane-association of mRNA decapping factors is independent of stress in budding yeast. *Sci. Rep*. **6**, 25477; doi: 10.1038/srep25477 (2016).

## Figures and Tables

**Table 1:** Yeast strains used in this work.

Strain		
(yTN)	Genotype	Reference
29	MATα, *leu2–3,112, trp1, ura3–52, cup1::LEU2/PGK1pG/MFA2pG*	62
34	MATa, *his3-∆1, leu2∆0, met15∆0, ura3∆0*	63
56	MATa, *his3∆1, leu2∆0, met15∆0, ura3∆0, DCP2-GFP (HIS3)*	64
57	MATa, *his3∆1, leu2∆0, met15∆0, ura3∆0, DHH1-GFP (HIS3)*	64
59	MATa, *his3∆1, leu2∆0, met15∆0, ura3∆0, LSM1-GFP (HIS3)*	64
82	MATa, *his3∆1, leu2∆0, met15∆0, ura3∆0, DCP1-TAP (HIS3)*	65
83	MATa, *his3∆1, leu2∆0, met15∆0, ura3∆0, PGK1-TAP (HIS3)*	64
100	MATa, *his3∆1, leu2∆0, met15∆0, ura3∆0, ANP1-GFP (HIS3)*	64
103	MATa, *his3∆1, leu2∆0, met15∆0, ura3∆0, SEC7-GFP (HIS3)*	64
122	MATa, *his3∆1, leu2∆0, met15∆0, ura3∆0, DPM1-TAP (HIS3)*	65
125	MATa, *his3∆1, leu2∆0, met15∆0, ura3∆0, SEC7-TAP (HIS3)*	65
193	MATa*, his3∆1, leu2∆0, met15∆0, ura3∆0, DCP2-TAP (HIS3)*	65
194	MATa, *his3∆1, leu2∆0, met15∆0, ura3∆0, POP2-TAP (HIS3)*	65
195	MATa, *his3∆1, leu2∆0, met15∆0, ura3∆0, DHH1-TAP (HIS3)*	65
197	MATa, *his3∆1, leu2∆0, met15∆0, ura3∆0, KAR2-TAP (HIS3)*	65
439	MATa, *his3∆1, leu2∆0, met15∆0, ura3∆0, DCP2-GFP (HIS3)*	This Work
*TRP1::HDEL-dsRED(NatMX)*		
441	MATa, *his3∆1, leu2∆0, met15∆0, ura3∆0, LSM1-GFP (HIS3)*	This Work
*TRP1::HDEL-dsRED(NatMX)*		
443	MATa, *his3∆1, leu2∆0, met15∆0, ura3∆0, DHH1-GFP (HIS3)*	This Work
*TRP1::HDEL-dsRED(NatMX)*		

## References

[b1] Parker R. RNA Degradation in Saccharomyces cerevisae. Genetics 191, 671–702 (2012). 22785621 10.1534/genetics.111.137265 PMC3389967

[b2] Balagopal V., Fluch L. & Nissan T. Ways and means of eukaryotic mRNA decay. Biochim Biophys Acta 1819, 593–603 (2012). 22266130 10.1016/j.bbagrm.2012.01.001

[b3] Nissan T., Rajyaguru P., She M., Song H. & Parker R. Decapping activators in Saccharomyces cerevisiae act by multiple mechanisms. Mol Cell 39, 773–783 (2010). 20832728 10.1016/j.molcel.2010.08.025 PMC2946179

[b4] Sheth U. & Parker R. Decapping and decay of messenger RNA occur in cytoplasmic processing bodies. Science 300, 805–808 (2003). 12730603 10.1126/science.1082320 PMC1876714

[b5] Hu W., Sweet T. J., Chamnongpol S., Baker K. E. & Coller J. Co-translational mRNA decay in Saccharomyces cerevisiae. Nature 461, 225–229 (2009). 19701183 10.1038/nature08265 PMC2745705

[b6] Pelechano V., Wei W. & Steinmetz L. M. Widespread Co-translational RNA Decay Reveals Ribosome Dynamics. Cell 161, 1400–1412 (2015). 26046441 10.1016/j.cell.2015.05.008 PMC4461875

[b7] Mangus D. A. & Jacobson A. Linking mRNA turnover and translation: assessing the polyribosomal association of mRNA decay factors and degradative intermediates. Methods 17, 28–37 (1999). 10075880 10.1006/meth.1998.0704

[b8] Li Z. . Rational extension of the ribosome biogenesis pathway using network-guided genetics. 7, e1000213 (2009). 10.1371/journal.pbio.1000213 PMC2749941 19806183

[b9] Huang L. . Mitochondria associate with P-bodies and modulate microRNA-mediated RNA interference. J Biol Chem 286, 24219–24230 (2011). 21576251 10.1074/jbc.M111.240259 PMC3129203

[b10] Cougot N., Cavalier A., Thomas D. & Gillet R. The dual organization of P-bodies revealed by immunoelectron microscopy and electron tomography. J Mol Biol 420, 17–28 (2012). 22484175 10.1016/j.jmb.2012.03.027

[b11] Stalder L. & Mühlemann O. Processing bodies are not required for mammalian nonsense-mediated mRNA decay. Rna 15, 1265–1273 (2009). 19474145 10.1261/rna.1672509 PMC2704072

[b12] Gibbings D. J., Ciaudo C., Erhardt M. & Voinnet O. Multivesicular bodies associate with components of miRNA effector complexes and modulate miRNA activity. Nat Cell Biol 11, 1143–1149 (2009). 19684575 10.1038/ncb1929

[b13] Wilhelm J. E., Buszczak M. & Sayles S. Efficient protein trafficking requires trailer hitch, a component of a ribonucleoprotein complex localized to the ER in Drosophila. Dev Cell 9, 675–685 (2005). 16256742 10.1016/j.devcel.2005.09.015

[b14] Kilchert C., Weidner J., Prescianotto-Baschong C. & Spang A. Defects in the secretory pathway and high Ca2+induce multiple P-bodies. Mol Biol Cell 21, 2624–2638 (2010). 20519435 10.1091/mbc.E10-02-0099 PMC2912349

[b15] Beckham C. J. . Interactions between brome mosaic virus RNAs and cytoplasmic processing bodies. J. Virol. 81, 9759–9768 (2007). 17609284 10.1128/JVI.00844-07 PMC2045432

[b16] Vergés E., Colomina N., Garí E., Gallego C. & Aldea M. Cyclin Cln3 is retained at the ER and released by the J chaperone Ydj1 in late G1 to trigger cell cycle entry. Mol Cell 26, 649–662 (2007). 17560371 10.1016/j.molcel.2007.04.023

[b17] Holmes K. J., Klass D. M., Guiney E. L. & Cyert M. S. Whi3, an S. cerevisiae RNA-binding protein, is a component of stress granules that regulates levels of its target mRNAs. PLos One 8, e84060 (2013). 24386330 10.1371/journal.pone.0084060 PMC3873981

[b18] Zhang H. . RNA Controls PolyQ Protein Phase Transitions. Mol Cell 60, 220–230 (2015). 26474065 10.1016/j.molcel.2015.09.017 PMC5221516

[b19] Toretsky J. A. & Wright P. E. Assemblages: functional units formed by cellular phase separation. J Cell Biol 206, 579–588 (2014). 25179628 10.1083/jcb.201404124 PMC4151146

[b20] Decker C. J., Teixeira D. & Parker R. Edc3p and a glutamine/asparagine-rich domain of Lsm4p function in processing body assembly in Saccharomyces cerevisiae. J Cell Biol 179, 437–449 (2007). 17984320 10.1083/jcb.200704147 PMC2064791

[b21] Jonas S. & Izaurralde E. The role of disordered protein regions in the assembly of decapping complexes and RNP granules. Genes Dev 27, 2628–2641 (2013). 24352420 10.1101/gad.227843.113 PMC3877753

[b22] Lin Y., Protter D. S. W., Rosen M. K. & Parker R. Formation and Maturation of Phase-Separated Liquid Droplets by RNA-Binding Proteins. Mol Cell 60, 208–219 (2015). 26412307 10.1016/j.molcel.2015.08.018 PMC4609299

[b23] Burke K. A., Janke A. M., Rhine C. L. & Fawzi N. L. Residue-by-Residue View of *in vitro* FUS Granules that Bind the C-Terminal Domain of RNA Polymerase II. Mol Cell 60, 231–241 (2015). 26455390 10.1016/j.molcel.2015.09.006 PMC4609301

[b24] Kroschwald S. . Promiscuous interactions and protein disaggregases determine the material state of stress-inducible RNP granules. Elife 4, e06807 (2015). 26238190 10.7554/eLife.06807 PMC4522596

[b25] Teixeira D., Sheth U., Valencia-Sanchez M. A., Brengues M. & Parker R. Processing bodies require RNA for assembly and contain nontranslating mRNAs. Rna 11, 371–382 (2005). 15703442 10.1261/rna.7258505 PMC1370727

[b26] Rieder S. E. & Emr S. D. Isolation of subcellular fractions from the yeast Saccharomyces cerevisiae. Curr Protoc Cell Biol 3.8–3.8.68, doi: 10.1002/0471143030.cb0308s08 (2001). 18228360

[b27] Rubin G. M. Preparation of RNA and ribosomes from yeast. Methods Cell Biol. 12, 45–64 (1975). 1105073 10.1016/s0091-679x(08)60951-6

[b28] Sweet T., Kovalak C. & Coller J. The DEAD-Box Protein Dhh1 Promotes Decapping by Slowing Ribosome Movement. 10, e1001342 (2012). 10.1371/journal.pbio.1001342 PMC3373615 22719226

[b29] Jagannathan S., Nwosu C. & Nicchitta C. V. Analyzing mRNA Localization to the Endoplasmic Reticulum via Cell Fractionation. Methods Mol Biol 714, 301–321 (2011). 21431749 10.1007/978-1-61779-005-8_19 PMC3718476

[b30] Yoon J.-H., Choi E.-J. & Parker R. Dcp2 phosphorylation by Ste20 modulates stress granule assembly and mRNA decay in Saccharomyces cerevisiae. J Cell Biol 189, 813–827 (2010). 20513766 10.1083/jcb.200912019 PMC2878948

[b31] Spirin A. S. The second Sir Hans Krebs Lecture. Informosomes. Eur J Biochem 10, 20–35 (1969). 4899460 10.1111/j.1432-1033.1969.tb00651.x

[b32] Stalder L. . The rough endoplasmatic reticulum is a central nucleation site of siRNA-mediated RNA silencing. EMBO J 32, 1115–1127 (2013). 23511973 10.1038/emboj.2013.52 PMC3630355

[b33] Rieder S. E. & Emr S. D. Overview of subcellular fractionation procedures for the yeast Saccharomyces cerevisiae. Curr Protoc Cell Biol 3.7–3.7.25, doi: 10.1002/0471143030.cb0307s07 (2001). 18228359

[b34] Becker A. H., Oh E., Weissman J. S., Kramer G. & Bukau B. Selective ribosome profiling as a tool for studying the interaction of chaperones and targeting factors with nascent polypeptide chains and ribosomes. Nat Protoc 8, 2212–2239 (2013). 24136347 10.1038/nprot.2013.133 PMC4001812

[b35] Gavin A.-C. . Proteome survey reveals modularity of the yeast cell machinery. Nature 440, 631–636 (2006). 16429126 10.1038/nature04532

[b36] Sweet T. J., Boyer B., Hu W., Baker K. E. & Coller J. Microtubule disruption stimulates P-body formation. Rna 13, 493–502 (2007). 17307817 10.1261/rna.355807 PMC1831866

[b37] Hesketh J. E. & Pryme I. F. Interaction between mRNA, ribosomes and the cytoskeleton. Biochem J 277 (Pt 1), 1–10 (1991). 1854327 10.1042/bj2770001 PMC1151183

[b38] Scopes R. K. Protein Purification. (Springer Science & Business Media, 2013).

[b39] Kuhn K. M., DeRisi J. L., Brown P. O. & Sarnow P. Global and specific translational regulation in the genomic response of Saccharomyces cerevisiae to a rapid transfer from a fermentable to a nonfermentable carbon source. Molecular and Cellular Biology 21, 916–927 (2001). 11154278 10.1128/MCB.21.3.916-927.2001 PMC86682

[b40] Ashe M. P., De Long S. K. & Sachs A. B. Glucose depletion rapidly inhibits translation initiation in yeast. Mol Biol Cell 11, 833–848 (2000). 10712503 10.1091/mbc.11.3.833 PMC14814

[b41] Uesono Y. & Toh-E A. Transient inhibition of translation initiation by osmotic stress. J Biol Chem 277, 13848–13855 (2002). 11796711 10.1074/jbc.M108848200

[b42] Huch S. & Nissan T. Interrelations between translation and general mRNA degradation in yeast. Wiley Interdiscip Rev RNA 5, 747–763 (2014). 24944158 10.1002/wrna.1244 PMC4285117

[b43] Holmes L. E. A., Campbell S. G., De Long S. K., Sachs A. B. & Ashe M. P. Loss of translational control in yeast compromised for the major mRNA decay pathway. Molecular and Cellular Biology 24, 2998–3010 (2004). 15024087 10.1128/MCB.24.7.2998-3010.2004 PMC371117

[b44] Brengues M., Teixeira D. & Parker R. Movement of eukaryotic mRNAs between polysomes and cytoplasmic processing bodies. Science 310, 486–489 (2005). 16141371 10.1126/science.1115791 PMC1863069

[b45] van Hoof A., Lennertz P. & Parker R. Yeast exosome mutants accumulate 3′-extended polyadenylated forms of U4 small nuclear RNA and small nucleolar RNAs. Molecular and Cellular Biology 20, 441–452 (2000). 10611222 10.1128/mcb.20.2.441-452.2000 PMC85098

[b46] LaCava J. . RNA degradation by the exosome is promoted by a nuclear polyadenylation complex. Cell 121, 713–724 (2005). 15935758 10.1016/j.cell.2005.04.029

[b47] Kadaba S., Wang X. & Anderson J. T. Nuclear RNA surveillance in Saccharomyces cerevisiae: Trf4p-dependent polyadenylation of nascent hypomethylated tRNA and an aberrant form of 5S rRNA. Rna 12, 508–521 (2006). 16431988 10.1261/rna.2305406 PMC1383588

[b48] Wang X. . Brome mosaic virus 1a nucleoside triphosphatase/helicase domain plays crucial roles in recruiting RNA replication templates. J. Virol. 79, 13747–13758 (2005). 16227294 10.1128/JVI.79.21.13747-13758.2005 PMC1262622

[b49] Boon den J. A., Chen J. & Ahlquist P. Identification of sequences in Brome mosaic virus replicase protein 1a that mediate association with endoplasmic reticulum membranes. J. Virol. 75, 12370–12381 (2001). 11711627 10.1128/JVI.75.24.12370-12381.2001 PMC116133

[b50] Frazier A. E. . Pam16 has an essential role in the mitochondrial protein import motor. Nat Struct Mol Biol 11, 226–233 (2004). 14981507 10.1038/nsmb735

[b51] Madrid A. S., Mancuso J., Cande W. Z. & Weis K. The role of the integral membrane nucleoporins Ndc1p and Pom152p in nuclear pore complex assembly and function. J Cell Biol 173, 361–371 (2006). 16682526 10.1083/jcb.200506199 PMC2063837

[b52] Bandyra K. J. & Luisi B. F. Licensing and due process in the turnover of bacterial RNA. RNA Biol 10, 627–635 (2013). 23580162 10.4161/rna.24393 PMC3710370

[b53] Khemici V., Poljak L., Luisi B. F. & Carpousis A. J. The RNase E of Escherichia coli is a membrane-binding protein. Mol Microbiol 70, 799–813 (2008). 18976283 10.1111/j.1365-2958.2008.06454.x PMC7610891

[b54] Strahl H. . Membrane recognition and dynamics of the RNA degradosome. PLos Genet 11, e1004961 (2015). 25647427 10.1371/journal.pgen.1004961 PMC4372235

[b55] Shahbabian K., Jamalli A., Zig L. & Putzer H. RNase Y, a novel endoribonuclease, initiates riboswitch turnover in Bacillus subtilis. EMBO J 28, 3523–3533 (2009). 19779461 10.1038/emboj.2009.283 PMC2782095

[b56] Hunt A., Rawlins J. P., Thomaides H. B. & Errington J. Functional analysis of 11 putative essential genes in Bacillus subtilis. Microbiology (Reading, Engl.) 152, 2895–2907 (2006). 10.1099/mic.0.29152-0 17005971

[b57] Jouannet V. . Cytoplasmic Arabidopsis AGO7 accumulates in membrane-associated siRNA bodies and is required for ta-siRNA biogenesis. EMBO J 31, 1704–1713 (2012). 22327216 10.1038/emboj.2012.20 PMC3321200

[b58] Lee Y. S. . Silencing by small RNAs is linked to endosomal trafficking. Nat Cell Biol 11, 1150–1156 (2009). 19684574 10.1038/ncb1930 PMC2737091

[b59] Schindelin J. . Fiji: an open-source platform for biological-image analysis. Nat Methods 9, 676–682 (2012). 22743772 10.1038/nmeth.2019 PMC3855844

[b60] Nissan T. & Parker R. Analyzing P-bodies in Saccharomyces cerevisiae. Meth Enzymol 448, 507–520 (2008). 19111192 10.1016/S0076-6879(08)02625-6 PMC2693489

[b61] Coller J. & Parker R. General translational repression by activators of mRNA decapping. Cell 122, 875–886 (2005). 16179257 10.1016/j.cell.2005.07.012 PMC1853273

[b62] Hatfield L., Beelman C. A., Stevens A. & Parker R. Mutations in trans-acting factors affecting mRNA decapping in Saccharomyces cerevisiae. Molecular and Cellular Biology 16, 5830–5838 (1996). 8816497 10.1128/mcb.16.10.5830 PMC231584

[b63] Brachmann C. B. . Designer deletion strains derived from Saccharomyces cerevisiae S288C: a useful set of strains and plasmids for PCR-mediated gene disruption and other applications. Yeast 14, 115–132 (1998). 9483801 10.1002/(SICI)1097-0061(19980130)14:2<115::AID-YEA204>3.0.CO;2-2

[b64] Huh W.-K. . Global analysis of protein localization in budding yeast. Nat Cell Biol 425, 686–691 (2003). 10.1038/nature02026 14562095

[b65] Ghaemmaghami S. . Global analysis of protein expression in yeast. Nat Cell Biol 425, 737–741 (2003). 10.1038/nature02046 14562106

